# Effect of ethnicity on care pathway and outcomes in patients hospitalized with influenza A(H1N1)pdm09 in the UK

**DOI:** 10.1017/S0950268814001873

**Published:** 2014-08-01

**Authors:** G. A. NYLAND, B. C. McKENZIE, P. R. MYLES, M. G. SEMPLE, W. S. LIM, P. J. M. OPENSHAW, R. C. READ, B. L. TAYLOR, S. J. BRETT, J. McMENAMIN, J. E. ENSTONE, B. BANNISTER, K. G. NICHOLSON, J. S. NGUYEN-VAN-TAM

**Affiliations:** 1 University of Nottingham, Nottingham, UK; 2 University of Liverpool, Liverpool, UK; 3 Nottingham University Hospitals NHS Trust, Nottingham, UK; 4 Imperial College, London, UK; 5 University of Southampton, Southampton, UK; 6 Portsmouth Hospitals NHS Trust, Portsmouth, UK; 7 Imperial College Healthcare NHS Trust, London, UK; 8 Health Protection Scotland, Glasgow, UK; 9 Department of Health, London, UK; 10 Royal Free London NHS Trust, London, UK; 11 University Hospitals of Leicester NHS Trust, Leicester, UK

**Keywords:** Epidemiology, influenza, influenza A, pandemic

## Abstract

Data were extracted from the case records of UK patients admitted with laboratory-confirmed influenza A(H1N1)pdm09. White and non-White patients were characterized by age, sex, socioeconomic status, pandemic wave and indicators of pre-morbid health status. Logistic regression examined differences by ethnicity in patient characteristics, care pathway and clinical outcomes; multivariable models controlled for potential confounders. Whites (*n* = 630) and non-Whites (*n* = 510) differed by age, socioeconomic status, pandemic wave of admission, pregnancy, recorded obesity, previous and current smoking, and presence of chronic obstructive pulmonary disease. After adjustment for *a priori* confounders non-Whites were less likely to have received pre-admission antibiotics [adjusted odds ratio (aOR) 0·43, 95% confidence interval (CI) 0·28–0·68, *P* < 0·001) but more likely to receive antiviral drugs as in-patients (aOR 1·53, 95% CI 1·08–2·18, *P* = 0·018). However, there were no significant differences by ethnicity in delayed admission, severity at presentation for admission, or likelihood of severe outcome.

## INTRODUCTION

Ethnicity (commonly regarded as partially synonymous with race) may evoke a strong sense of identity, unity or difference – factors inextricably linked to health. There are grounds for postulating ethnic differences in exposure to influenza virus, susceptibility to infection once exposed, and timely access to effective treatment [1, 2]. Disparities or inequalities in influenza-related outcomes such as pneumonia can be observed for some minority groups [3, 4]. A cross-sectional survey of 1479 US households (with oversampling of minority groups) suggests Spanish-speaking Hispanics were at increased risk of presumed influenza A(H1N1)pdm09 exposure, but reduced risk of related complications; Blacks were more susceptible to self-reported complications [5]. Analysis of US Centers for Disease Control and Prevention (CDC) surveillance data collected during the 2009 pandemic found no ethnic/racial disparities in healthcare-seeking behaviour for influenza-like illness, but higher hospitalization rates in minority groups and higher paediatric mortality in Hispanics [6]. A Canadian study of 413 laboratory-confirmed influenza A(H1N1)pdm09 cases compared to test-negative community controls reports over-representation of ethnic minority cases [7].

These seemingly consistent patterns are concerning and while the effect of ethnicity may have plausible biological underpinnings it is also strongly linked to social context, requiring that international comparisons be made with caution. In the UK, based on a total of 70 paediatric deaths related to influenza A(H1N1)pdm09 (including those in the community), age-standardized mortality rates for Bangladeshi children [47 deaths per million population (dpm), 95% confidence interval (CI) 17–103] and Pakistani children (36 dpm, 95% CI 18–64) were found to be higher than for White British children (4 dpm, 95% CI 3–6) [8]. Where such inequitable outcomes are suggested, our professional and moral obligations to redress them, reduce disparities in access to healthcare and improve patients’ experiences can only be effected through measuring all relevant dimensions – one of which is ethnicity [9].

**Fig. 1.**
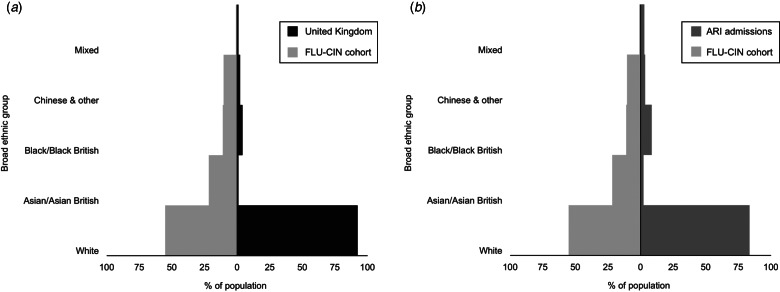
FLU-CIN population pyramids for ethnic composition* with comparison to (*a*) the UK general population† and (*b*) admissions to UK hospitals with acute respiratory infection (ARI) in the pre-pandemic period‡. [* 1140 cases; excludes 380 cases (25%) missing ethnicity data. † Demographic data on ethnicity derived from Office of National Statistics Census (2001) and General Register Office for Scotland and Northern Ireland Statistics and Research Agency (2001). ‡ Hospital Episodes Statistics data on primary discharge codes relating to possible influenza admissions (J06, J10, J11, J13–22) during November 2008–March 2009.]

**Fig. 2.**
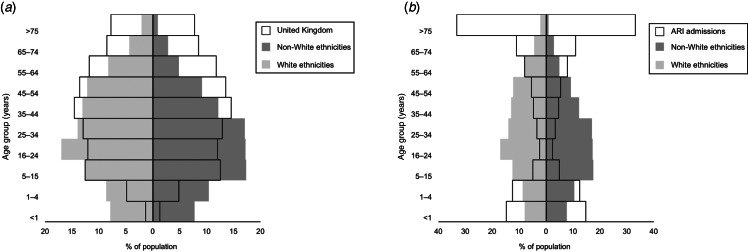
FLU-CIN population pyramids for age by broad ethnic group* with comparison to (*a*) the UK general population† and (*b*) admissions to UK hospitals with acute respiratory infection in the pre-pandemic period‡. [* 1140 cases; excludes 380 cases (25%) missing ethnicity data. † Demographic data on age distribution derived from Office of National Statistics 2009 mid-year population estimates (www.statistics.gov.uk). ‡ Hospital Episodes Statistics data on primary discharge codes relating to possible influenza admissions (J06, J10, J11, J13–22) during November 2008–March 2009.]

During the 2009 influenza pandemic the Department of Health (England) collected data on risk factors, including ethnicity, via the Influenza Clinical Information Network (FLU-CIN) programme [10]. Analysis of the first-wave FLU-CIN cohort (May–September 2009) indicated that ethnic minorities were over-represented among those admitted to hospital [10]; this was less pronounced in the second wave [11], but nevertheless raises the possibility of systematic differences in care. Figures 1 and 2, respectively, compare the ethnic composition and age profile of the FLU-CIN cohort with that of the UK general population and persons admitted to UK hospitals with acute respiratory infection (ARI) in the immediate pre-pandemic period winter season. To gain insight into these striking observations we report an analysis of FLU-CIN enhanced surveillance data over both pandemic waves, investigating possible ethnic differences in care pathway and clinical outcome in patients hospitalized with laboratory-confirmed influenza A(H1N1)pdm09 in the UK.

## METHODS

As previously described, trained nursing staff extracted data from the case records of patients hospitalized for laboratory-confirmed influenza A(H1N1)pdm09, without other selection criteria [10]. These included ethnicity as recorded in case notes, according to the Office for National Statistics Census classification (www.statistics.gov.uk). This official UK system of classification can be regarded as equivalent to racial group in the majority of cases.

We conceptualized a patient care pathway commencing with pre-admission care in the community; primary healthcare access indicators comprised use of self-medication, general practitioner (GP) consultation and receipt of pre-admission antibiotics or antivirals. Secondary healthcare access indicators comprised two proxy measures of access to hospital: admission delay of ⩾4 days from illness onset and illness severity at presentation for admission. The pathway concluded with indicators corresponding to in-patient care, these being receipt of in-patient antibiotics or antivirals. The clinical outcomes of interest were length of stay in hospital, admission to high-dependency or critical-care facilities and death. A number of covariates related to this pathway and clinical outcomes were identified and defined as follows.

Socioeconomic status was estimated from postcode of residence using the English Index of Multiple Deprivation (IMD) 2007, a composite area-based measure of deprivation that takes account of income, employment, health status and disability, education and skills, access to services, living environment and area-level crime [12]. IMD scores were grouped to facilitate comparison by ‘most affluent’ (IMD ⩽14·999), ‘affluent’ (IMD 15–29·999), ‘deprived’ (IMD 30–44·999) and ‘most deprived’ (IMD ⩾45) status.

National surveillance data determined assignment to the first (pre-September 2009) or second pandemic wave [13]. The National Pandemic Flu Service (NPFS) antiviral distribution system began operation during the first wave on 23 July 2009 [14]; we created a variable representing availability of the NPFS in order to model the effect of access to the service on antiviral availability.

Baseline health status incorporated a measure of the presence of comorbidities, the Charlson Comorbidity Index (CCI); the weighted CCI scores were categorized as ‘0’ (no comorbidities), 1–2, 3–5 and >5 [15, 16].

Early treatment with antivirals was defined as receipt within 2 days of symptom onset; late treatment was defined as receipt >2 days after onset of symptoms.

Delayed admission was defined by pandemic influenza experts on the FLU-CIN Steering Group as admission delay of ⩾4 days following onset of symptoms.

Illness severity at presentation was assessed using the community assessment tools (CATs) for triage (seven criteria as outlined in Supplementary Table S1), as recommended for use during a severe pandemic by the Department of Health, England [17, 18].

Admission for ⩾2 days was regarded as prolonged and thus a proxy measure for a more severe illness during hospitalization.

Type of admission was coded as level 0: patients whose care needs can be met through normal ward care; level 1: patients at risk of deteriorating or recently relocated from higher levels of care whose needs can be met on an acute ward with additional advice and support from the critical care team; level 2: patients requiring more detailed observation or intervention including support for a single failing organ system and those ‘stepping down’ from higher levels of care – high dependency unit (HDU); level 3: patients requiring advanced respiratory support alone or basic respiratory support together with support of at least two organ systems. This includes all complex patients requiring support for multi-organ failure – intensive care unit (ICU).

Univariate logistic regression analyses (Table 1) examined unadjusted associations between ethnicity (White or non-White) and patient characteristics at the point of admission to hospital with influenza A(H1N1)pdm09. A multivariable logistic regression model (Table 2) was used to adjust care pathway and clinical outcome indicators for potential confounders including age, sex, socioeconomic status, pandemic wave and (for outcomes related to in-patient care and mortality) delayed admission and severity at presentation for admission (model A). An alternative conceptual model (model B) further adjusted for variables found to be significantly maldistributed according to ethnicity (recorded obesity, current smoking and chronic obstructive pulmonary disease; pregnancy was excluded owing to the inclusion of males in the cohort).

**Table 1.** FLU-CIN patients’ characteristics at the point of admission to hospital with influenza A(H1N1)pdm09 in the UK by broad ethnic group (n = 1140)

Characteristics	White (*N* = 630), *n* (%)	Non-White (*N* = 510), *n* (%)	Crude OR (95% CI)	*P* value
Ethnic grouping, *N*/*n* (%)				
White	630/1140 (55·3)			
Mixed		11/1140 (1·0)		
Asian or Asian British		249/1140 (21·8)		
Black or Black British		129/1140 (11·3)		
Chinese and other		121/1140 (10·6)		
				
Sex				
Male	291 (46·2)	238 (46·7)	1·00	
Female	339 (53·8)	272 (53·3)	0·98 (0·78–1·24)	0·873
Age (years)				
<1	50 (7·9)	40 (7·8)	1·00	***P* trend = 0·001**
1–4	55 (8·7)	53 (10·4)	1·20 (0·69–2·11)	
5–15	79 (12·5)	89 (17·5)	1·41 (0·84–2·36)	
16–24	107 (17·0)	88 (17·3)	1·03 (0·62–1·70)	
25–34	88 (14·0)	87 (17·1)	1·24 (0·74–2·06)	
35–44	82 (13·0)	62 (12·2)	0·95 (0·56–1·61)	
45–54	77 (12·2)	47 (9·2)	0·76 (0·44–1·32)	
55–64	52 (8·3)	25 (4·9)	0·60 (0·32–1·13)	
65–74	27 (4·3)	14 (2·8)	0·65 (0·30–1·40)	
>75	13 (2·1)	5 (1·0)	0·48 (0·16–1·46)	
Socioeconomic status (IMD group)				
Most affluent (IMD ⩽14·999)	92 (14·6)	35 (6·9)	1·00	***P* trend < 0·001**
Affluent (IMD 15–29·999)	126 (20·0)	113 (22·2)	2·36 (1·48–3·75)	
Deprived (IMD 30–44·999)	59 (9·4)	134 (26·3)	5·97 (3·64–9·80)	
Most deprived (IMD ⩾45)	91 (14·4)	143 (28·0)	4·13 (2·58–6·61)	
Missing data	262 (41·6)	85 (16·7)		
Pandemic wave				
First wave	180 (28·6)	337 (66·1)	1·00	
Second wave	450 (71·4)	173 (33·9)	0·21 (0·16–0·26)	**<0·001**
Health status				
Current pregnancy*	27 (16·0)	39 (26·0)	1·85 (1·07–3·20)	**0·029**
Recorded obesity†	26 (4·1)	10 (2·0)	0·46 (0·22–0·97)	**0·042**
Current smoker	120 (19·1)	44 (8·6)	0·45 (0·31–0·66)	**<0·001**
Missing data	220 (34·9)	229 (44·9)		
Ever smoked	171 (27·1)	64 (12·5)	0·41 (0·29–0·58)	**<0·001**
Missing data	220 (34·9)	229 (44·9)		
Asthma diagnosis	158 (25·1)	137 (26·9)	1·10 (0·84–1·43)	0·494
COPD diagnosis	49 (7·8)	8 (1·6)	0·19 (0·09–0·40)	<0·001
Other lung disease	15 (2·4)	13 (2·6)	1·07 (0·51–2·27)	0·855
No comorbidity, CCI 0	322 (51·1)	299 (58·6)	1·00	*P* trend = 0·052
CCI 1–2	272 (43·2)	181 (35·5)	0·72 (0·56–0·92)	
CCI 3–5	35 (5·6)	28 (5·5)	0·86 (0·51–1·45)	
CCI >5	1 (0·2)	2 (0·4)	2·15 (0·19–23·88)	

IMD, English Index of Multiple Deprivation (2007); COPD, chronic obstructive pulmonary disease; CCI, Charlson Comorbidity Index; OR, odds ratio; CI, confidence interval.

Percentages may not add up to 100 due to rounding; statistically significant results shown in bold (*P* < 0·05).

* Expressed as a percentage of women of childbearing age (14–44 years).

† Physician-recorded in case notes.

**Table 2.** Care pathway and clinical outcomes for patients admitted with influenza A(H1N1)pdm09 in the UK by broad ethnic group (n = 1140)

Variable	White (*N* = 630) *n* (%)	Non-White (*N* = 510) *n* (%)	Crude OR (95% CI); *P* value	Adjusted OR (95% CI); *P* value (model A)	Adjusted OR (95% CI); *P* value (model B)
Primary healthcare access indicators					
Self-medication	49 (7·8)	39 (7·7)	0·98 (0·63–1·52); 0·934	1·33 (0·75–2·35); 0·327	1·28 (0·72–2·28); 0·402
GP consultation	180 (28·6)	139 (27·3)	0·94 (0·72–1·22); 0·622	0·82 (0·58–1·15); 0·255	0·84 (0·59–1·19); 0·322
Missing data	296 (47·0)	227 (44·5)			
Pre-admission antibiotic	142 (22·5)	54 (10·6)	**0·41 (0·29–0·57); < 0·001**	**0·43 (0·28–0·68); <0·001**	**0·42 (0·27–0·67); <0·001**
Pre-admission antiviral	68 (10·8)	51 (10·0)	0·92 (0·63–1·35); 0·663	0·86 (0·51–1·43); 0·554	0·81(0·48–1·35); 0·412
Secondary healthcare access indicators					
Admission delay ⩾4 days	133 (21·1)	95 (18·6)	**0·65 (0·48–0·88); 0·006**	0·73 (0·49–1·09); 0·119	0·71 (0·47–1·07); 0·103
Missing data	198 (31·4)	86 (16·8)			
Severity at presentation†	468 (74·3)	367 (72·0)	0·89 (0·68–1·16); 0·378	1·10 (0·79 −1·54); 0·576	1·16 (0·83–1·64); 0·387
In-patient care indicators					
In-patient antibiotic	513 (81·4)	440 (86·3)	**1·43 (1·04–1·98); 0·029**	1·37* (0·84–2·22); 0·204	1·46 (0·94–2·26); 0·095
In-patient antiviral	452 (71·8)	398 (78·0)	**1·40 (1·07–1·84); 0·015**	1·53* **(1·08–2·18); 0·018**	**1·66 (1·15–2·39); 0·006**
Clinical outcomes					
Length of stay ⩾2 days	427 (67·8)	357 (70·0)	0·82 (0·61–1·11); 0·195	1·04* (0·71–1·53); 0·840	1·11 (0·75–1·62); 0·607
Missing data	93 (14·8)	41 (8·0)			
Level 2 or 3 admission‡	109 (17·3)	66 (12·9)	**0·71 (0·51–0·99); 0·043**	1·19* (0·74–1·92); 0·478	1·25 (0·77–2·03); 0·368
Death	35 (5·6)	20 (3·9)	0·69 (0·40–1·22); 0·203	0·80* (0·35–1·82); 0·602	0·77 (0·33–1·80); 0·550

GP, General practitioner; OR, odds ratio; CI, confidence interval; adjusted OR (model A), adjusted for *a priori* confounders of age, sex, English Index of Multiple Deprivation (IMD 2007) score derived from postal code of residence and pandemic wave; adjusted OR (model B), adjusted for age, sex, IMD 2007 score, pandemic wave, recorded obesity, current smoking and chronic obstructive pulmonary disease.

* Adjusted for admission delay ⩾4 days and severity at presentation for admission.

† One or more clinical indicators of severe disease at triage (see text).

‡ Requiring high-dependency unit or critical care unit.

Percentages may not add up to 100 due to rounding; statistically significant results shown in bold (*P* < 0·05).

### Ethical standards

The Ethics and Confidentiality Committee (ECC) of the National Information Governance Board for Health and Social Care (NIGB) gave permission for the collection of patient-identifiable data for FLU-CIN in May 2009, noting the urgency and wider public interest of this surveillance study. Section 251 approval was not sought for exemption from gaining informed consent; provision of patient information in sentinel centres was stipulated. The authors assert that all procedures contributing to this work comply with the ethical standards of the relevant national and institutional committees on human experimentation and with the Helsinki Declaration of 1975, as revised in 2008.

## RESULTS

Of 1520 admissions in the FLU-CIN cohort [11], 1140 (75·0%) had ethnicity recorded (missing data *n* = 380, 25·0%). A summary of patients’ characteristics is given in Table 1. Non-White patients constituted almost half (*n* = 510, 44·7%) of the study population, the largest subgroup being Asian or Asian British (*n* = 249, 21·8%). Similar data for major non-White ethnic subgroups are provided in Supplementary Table S2.

Table 2 presents unadjusted (crude) odds and adjusted odds for both logistic regression models comparing non-Whites to Whites; these are grouped by care pathway and clinical outcome indicators. Similar data for major non-White ethnic subgroups are provided in Supplementary Table S3.

In respect of access to primary healthcare non-Whites were less likely to receive pre-admission antibiotics [model A, adjusted aOR (aOR) 0·43, 95% CI 0·28–0·68, *P* < 0·001), but no more or less likely to receive pre-admission antivirals (aOR 0·86, 95% CI 0·51–1·43, *P* = 0·554). Substituting the *a priori* variable for pandemic wave in model A for a variable representing access to the NPFS did not significantly alter the likelihood of receiving pre-admission antivirals by ethnicity (aOR 0·89, 95% CI 0·54–1·47, *P* = 0·662). Furthermore, there was no significant difference in the interval between date of symptom onset and date of antiviral receipt (i.e. early *vs*. late treatment), by ethnicity (OR 1·27, 95% CI 0·90–1·77, *P* = 0·169). Insufficient data on pandemic vaccination were available for inclusion in multivariable models (missing data, 73·5%) and 750 cases presented for admission prior to the availability of vaccine.

As a proxy measure of access to secondary healthcare, crude odds indicated non-Whites were less likely to experience an admission delay of ⩾4 days after symptom onset, but after adjustment this difference was not statistically significant. Likewise, no significant difference in illness severity at presentation for admission was observed between Whites and non-Whites (Table 2).

During the in-patient phase of the pathway non-White ethnic groups were more likely to receive antiviral drugs (model A: aOR 1·53, 95% CI 1·08–2·18, *P* = 0·018). A higher likelihood of non-Whites receiving antibiotics as in-patients became non-significant after adjustment.

In terms of clinical outcomes no significant differences were found for in-patient stays of ⩾2 days (indicating protracted illness). Crude odds indicated non-Whites were less likely to require high-dependency or intensive-care unit admission; however, after adjustment this difference was not statistically significant. No significant differences were found for mortality.

## DISCUSSION

Poorer influenza-related outcomes among indigenous peoples have been observed in Alaska, Sierra Leone [19], Australia [20] and New Zealand [4, 21] during previous influenza pandemics. Similar observations were made among Canadian First Nations communities [22] and in Alaska [23] during the 2009 pandemic. By contrast, the UK's ethnic minority population is non-indigenous, and has resulted largely from immigration over the last 60 years. However, larger non-indigenous ethnic groups in North America were also reported to experience adverse outcomes during the 2009 pandemic [7, 24]. We can therefore infer complex relationships between biological and social factors that shape observed differences by ethnicity. For this reason it may not be prudent to generalize findings from one country to the ethnic minorities of another.

Measuring ethnicity accurately is a further challenge, and the value of aggregate ethnicity data in epidemiological research is contentious. Individuals may define their ethnicity in terms of parentage, race, cultural heritage or affiliation (for example). As race, skin colour, country of origin or cultural affiliation are not necessarily synonymous with a particular ethnic risk profile, there is some potential for misclassification bias. Broad groupings such as ‘White’ or ‘non-White’ encompass much diversity (in social, cultural, religious, or genetic traits for example) and sensitivities to being so ‘lumped together’ must be acknowledged. Such aggregation has pragmatic value (overcoming low numbers encountered with more granular ethnic groupings, where annual data cannot be pooled [25]), precedent (usage in routine statistics) and utility in hypothesis generation to scrutinize access inequalities. Unfortunately, although aggregation does improve statistical confidence, it also increases heterogeneity. As 45% of the FLU-CIN cohort was non-White, in order to balance these considerations we also compared major non-White ethnic subgroups to White groups (Supplementary Tables S2 and S3).

The pattern of over-representation of non-Whites in both the FLU-CIN cohort and the population admitted with ARI in 2008–2009 is broadly consistent (Fig. 1). ARI offers a valid comparison given that admission thresholds by ethnicity are unlikely to differ between presentation with pandemic influenza or with clinically indistinguishable respiratory virus infections (including seasonal influenza) in the previous winter season. It is equally important to note the highly significant reversal in proportion of Whites to non-Whites in FLU-CIN cases between the first and second pandemic waves (Table 1). Mapping of cases by postcode of residence according to pandemic wave (Supplementary Fig. S1) highlighted urban clustering of admissions, particularly around London and the West Midlands during the first wave, spreading to the East Midlands and Northwest (with less ethnically diverse populations) during the second wave. These data suggest that the initial predominance of non-White patients in the first pandemic wave and some degree of reversal as pandemic activity dispersed in the second wave was mainly attributable to place of residence, rather than inherent predisposition. This explanation would not exclude the possibility that vulnerable members of ethnic minority communities experienced higher risk of exposure to influenza A(H1N1)pdm09 prior to availability of pandemic vaccine, which may still indicate the need for more effective promotion of measures such as hand hygiene advice and social distancing during the initial pandemic period.

While non-Whites were less likely to have received pre-admission antibiotics, there was no compensatory increase in the likelihood of receiving pre-admission antivirals. If antibiotic prescriptions do reflect access to primary healthcare then use of fewer antibiotics for non-Whites might indicate a reduced propensity among minority groups to seek care, culturally or educationally mediated beliefs about the value of antibiotics, or typical attitudes to risk [1, 2]. Alternatively, it might indicate antibiotic prescribing practices that discriminate against ethnic minorities or are inappropriately responsive to demand from White patients. We note that Whites and non-Whites were equally likely to self-medicate; access barriers to community pharmacies may be lower. However, similar access to GP consultations and to pre-admission antivirals between Whites and non-Whites would argue against wider access barriers.

For access to secondary healthcare we found no difference between Whites and non-Whites in terms of admission delay or disease severity at presentation for admission (Table 2 and Supplementary Table S1), suggesting that differences in pre-hospital antibiotic receipt by ethnicity did not impact on timing of, or severity at admission. FLU-CIN was not configured to report influenza A(H1N1)pdm09 hospitalization rates by ethnicity for hospital catchment areas, thus our data cannot comment on excess rates observed among minority groups measured elsewhere [6, 22, 24, 26, 27].

Given the absence of any difference between Whites and non-Whites at the point of admission, it is somewhat surprising that the latter were more likely to have received antiviral drugs as in-patients (Table 2). This is not explained by non-Whites presenting disproportionately as first-wave cases, when prescribing of antivirals could have been more cautious. The difference by ethnicity was not significant for first-wave admissions, where 74·4% of Whites (*n* = 134) and 79·0% of non-Whites (*n* = 266) received in-patient antivirals (model A: aOR 1·23, 95% CI 0·73–2·07, *P* = 0·434). The difference was, however, significant for second-wave admissions, where 70·7% of Whites (*n* = 318) and 76·3% of non-Whites (*n* = 132) received in-patient antivirals (model A: aOR 1·76, 95% CI 1·05–2·94, *P* = 0·033).

Our study did not find any evidence that small differences in care pathway resulted in significant differences in clinical outcome by ethnicity. Non-Whites were not disadvantaged as judged by mortality or level 2/3 admission using either our a *priori* or conceptual model to adjust for potential confounding. Our results juxtapose the Canadian experience where indigenous First Nations ethnicity was an independent risk factor for ICU admission [28]. The UK non-White population may be regarded as non-indigenous, yet mortality in New Zealand was reported as ‘significantly’ higher in non-indigenous Pacific Peoples (infection rate-adjusted rate ratio 3·28, 95% CI 1·44–7·49, *P* value not stated) [27]. This may suggest that indigenous ethnic status, while being partly genetic/biological (perhaps modulating illness severity or response to therapy), is being confounded by other factors. Quinn and colleagues postulate the operation of differential risks of influenza exposure, susceptibility and healthcare access that worsen existing inequities [5], which may themselves be independently associated with ethnicity/race or socioeconomic status or both. In their review of 4874 influenza A(H1N1)pdm09-related discharges in Massachusetts, Placzek & Madoff found both minority ethnic/racial group and lower socioeconomic status predicted ICU stay [24]. Compared to non-Hispanic Whites, Hispanics were less likely to be admitted to an ICU (aOR 0·52, 95% CI 0·32–0·86, *P* < 0·05); of patients admitted to ICUs, 63% of Hispanics, 43% of non-Hispanic Blacks and only 13% of non-Hispanic Whites were among the least affluent socioeconomic group [24]. Although the authors were unable to measure differential access to healthcare, differences in risk perception, healthcare reform, cultural or language barriers are mooted as possible contributors. Taken together with our own findings, these observations likely reflect a complex and confounded relationship between clinical outcomes for influenza A(H1N1)pdm09, ethnicity and social disadvantage [29].

Our study does not provide compelling evidence of important disparities in ‘downstream’ access to primary or secondary care treatment for influenza A(H1N1)pdm09 in the UK. Our findings do, however, mirror the over-representation of non-Whites among A(H1N1)pdm09-related hospital admissions reported elsewhere. This might reflect a difference in susceptibility to infection, either biological or by means of difference in uptake of the ‘midstream’ intervention of vaccination. Our study refutes vaccination as an explanation, given that over-representation preceded availability of pandemic vaccine. Over-representation of non-Whites most likely relates to ‘upstream’ factors that determine exposure to the influenza virus. We provide evidence that geography played a key role in determining this pattern, which is also the basis of measuring socioeconomic status in the UK. Co-related upstream factors include population density, crowded living conditions, reliance on public transport, occupational group, acceptability of social distancing and use of communal childcare facilities [1, 2]. The challenge for future research is to unpick the contribution of such factors and to determine whether variation is attributable to ethnicity, socioeconomic status or both.

## CONCLUSIONS

Ethnicity was not a significant predictor of inequities or disparities in care pathway or of clinical outcomes for patients hospitalized with influenza A(H1N1)pdm09 in the UK. We did not find any evidence that small differences in care pathway resulted in significant differences in clinical outcome by ethnicity.

## Supplementary material

For supplementary material accompanying this paper visit http://dx.doi.org/10.1017/S0950268814001873. click here to view supplementary material
