# Supplementary material for: Effect of ethnicity on care pathway and outcomes in patients hospitalized with influenza A(H1N1)pdm09 in the UK
Source: Epidemiol Infect. 2014 Aug 1;143(6):1129–38. doi: 10.1017/S0950268814001873 (PMC4412072; doi:10.1017/S0950268814001873)
Supplement: Supplementary file 1 [file S0950268814001873sup.zip › S0950268814001873sup003.doc]

*Epidemiology and Infection*

Effect of ethnicity on care pathway and outcomes in patients hospitalized with influenza A(H1N1)pdm09 in the UK

G. A. Nyland, B. C. McKenzie, P. R. Myles, M. G. Semple, W. Shen Lim, P. J. M. Openshaw, R. C. Read, B. L. Taylor, S. J. Brett, J. McMenamin, J. E. Enstone, B. Bannister, K. G. Nicholson, J. S. Nguyen-Van-Tam, on behalf of the Influenza Clinical Information Network (FLU-CIN)

Supplementary Material

## Supplementary Table S2 – FLU-CIN patient characteristics at the point of admission to hospital with influenza A(H1N1)pdm09 in the UK across major non-White ethnic sub-groups (n = 510)

IMD = English Index of Multiple Deprivation (2007); COPD = chronic obstructive pulmonary disease; CCI = Charlson Comorbidity Index; OR = odds ratio; CI = confidence interval; *Expressed as a percentage of women of childbearing age (14 – 44 years); †Physician recorded in case notes; Percentages may not add to 100 due to rounding; Statistically significant results shown in bold (p<0.05) noting reference group = White^1^ & p for trend^2^; ‡Could not be calculated due to insufficient data

| Characteristics | Asian/Asian British  n = 249 (%) | Black/Black British  n = 129 (%) | Mixed, Chinese/Others  n = 132 (%) |
| --- | --- | --- | --- |
| **Sex** | | | |
| Male | 115 (46.2) | 58 (45.0) | 65 (49.2) |
| Female | 134 (53.8) | 71 (55.0) | 67 (50.8) |
| Crude OR^1^ (95% CI); p value | 1.00 (0.75 – 1.34); 0.999 | 1.05 (0.72 – 1.54); 0.799 | 0.88 (0.61 – 1.29); 0.523 |
| **Age (years)** | | | |
| <1 | 19 (7.6) | 7 (5.4) | 14 (10.6) |
| 1 – 4 | 23 (9.2) | 11 (8.5) | 19 (14.4) |
| 5 – 15 | 28 (11.2) | 41 (31.8) | 20 (15.2) |
| 16 – 24 | 46 (18.5) | 19 (14.7) | 23 (17.4) |
| 25 – 34 | 50 (20.1) | 18 (14.0) | 19 (14.4) |
| 35 – 44 | 30 (12.1) | 17 (13.2) | 15 (11.4) |
| 45 – 54 | 27 (10.8) | 9 (7.0) | 11 (8.3) |
| 55 – 64 | 15 (6.0) | 3 (2.3) | 7 (5.3) |
| 65 – 74 | 8 (3.2) | 3 (2.3) | 3 (2.3) |
| >75 | 3 (1.2) | 1 (0.8) | 1 (0.8) |
| Crude OR^1^ (95% CI); p value^2^ | 0.96 (0.90 – 1.03); 0.281 | **0.87 (0.80 – 0.95); 0.002** | **0.88 (0.81 – 0.96); 0.003** |

| Characteristics | Asian/Asian British  n = 249 (%) | | Black/Black British  n = 129 (%) | Mixed, Chinese/Others  n = 132 (%) | |
| --- | --- | --- | --- | --- | --- |
| **Socio-economic status (IMD group)** | | | | | |
| Most affluent (IMD ≤14.999) | 22 (8.8) | 5 (3.9) | | | 8 (6.1) |
| Affluent (IMD 15 – 29.999) | 56 (22.5) | 32 (24.8) | | | 25 (18.9) |
| Deprived (IMD 30 – 44.999) | 63 (25.3) | 46 (35.7) | | | 25 (18.9) |
| Most deprived (IMD ≥45) | 65 (26.1) | 32 (24.8) | | | 46 (34.9) |
| *Missing data* | 43 (17.3) | 14 (10.9) | | | 28 (21.2) |
| Crude OR^1^ (95% CI); p value^2^ | **1.44 (1.23 – 1.69); <0.001** | **1.57 (1.28 – 1.92); <0.001** | | | **1.71 (1.39 – 2.11); <0.001** |
| **Pandemic wave** | | | | | |
| First wave | 169 (67.9) | 105 (81.4) | | | 63 (47.7) |
| Second wave | 80 (32.1) | 24 (18.6) | | | 69 (52.3) |
| Crude OR^1^ (95% CI); p value | **0.19 (0.14 – 0.26); <0.001** | **0.09 (0.06 – 0.15); <0.001** | | | **0.44 (0.30 – 0.64); <0.001** |

*Continues…*

| Characteristics | Asian/Asian British  n = 249 (%) | | Black/Black British  n = 129 (%) | Mixed, Chinese/Others  n = 132 (%) | |
| --- | --- | --- | --- | --- | --- |
| **Health status** | | | | | |
| Current pregnancy* | 21 (27.3) | 9 (23.1) | | | 9 (26.5) |
| Crude OR^1^ (95% CI); p value | **1.97 (1.03 – 3.77); 0.040** | 1.58 (0.67 – 3.70); 0.294 | | | 1.89 (0.80 – 4.50); 0.149 |
| Recorded obesity† | 4 (1.6) | 5 (3.9) | | | 1 (0.8) |
| Crude OR^1^ (95% CI); p value | 0.38 (0.13 – 1.10); 0.074 | 0.94 (0.35 – 2.49); 0.896 | | | 0.18 (0.02 – 1.32); 0.09 |
| Current smoker | 17 (6.8) | 11 (8.5) | | | 16 (12.1) |
| Missing data | 118 (47.4) | 70 (54.3) | | | 41 (31.1) |
| Crude OR^1^ (95% CI); p value | **0.36 (0.21 – 0.63); <0.001** | 0.55 (0.28 – 1.10); 0.093 | | | **0.52 (0.29 – 0.92); 0.025** |
| Ever smoked | 24 (9.6) | 15 (11.6) | | | 25 (18.9) |
| Missing data | 118 (47.4) | 70 (54.3) | | | 41 (31.1) |
| Crude OR^1^ (95% CI); p value | **0.31 (0.19 – 0.51); <0.001** | **0.48 (0.26 – 0.88); 0.019** | | | **0.53 (0.32 – 0.87); 0.013** |
| Asthma diagnosis | 67 (26.9) | 43 (33.3) | | | 27 (20.5) |
| Crude OR^1^ (95% CI); p value | 1.10 (0.79 – 1.53); 0.576 | 1.49 (0.99 – 2.25); 0.054 | | | 0.77 (0.49 – 1.22); 0.261 |
| COPD diagnosis | 6 (2.4) | 0 (0.0) | | | 2 (1.5) |
| Crude OR^1^ (95% CI); p value | **0.29 (0.12 – 0.69); 0.005** | — ‡ | | | **0.18 (0.04 – 0.76); 0.019** |
| Other lung disease | 6 (2.4) | 5 (3.9) | | | 2 (1.5) |
| Crude OR^1^ (95% CI); p value | 1.01 (0.39 – 2.64); 0.980 | 1.65 (0.59 – 4.63); 0.339 | | | 0.63 (0.14 – 2.79); 0.544 |
| **Weighted co-morbidity burden: Charlson Comorbidity Index score (CCI)** | | | | | |
| 0 (no co-morbidity) | 137 (55.0) | 70 (54.3) | | | 92 (69.7) |
| 1 – 2 | 96 (38.6) | 53 (41.1) | | | 32 (24.2) |
| 3 – 5 | 15 (6.0) | 6 (4.7) | | | 7 (5.3) |
| >5 | 1 (0.4) | 0 (0.0) | | | 1 (0.8) |
| Crude OR^1^ (95% CI); p value^2^ | 0.85 (0.64 – 1.15); 0.296 | 0.88 (0.60 – 1.29); 0.514 | | | **0.45 (0.30 – 0.68); <0.001** |

## *End*
