# Supplementary material for: Effect of ethnicity on care pathway and outcomes in patients hospitalized with influenza A(H1N1)pdm09 in the UK
Source: Epidemiol Infect. 2014 Aug 1;143(6):1129–38. doi: 10.1017/S0950268814001873 (PMC4412072; doi:10.1017/S0950268814001873)
Supplement: Supplementary file 1 [file S0950268814001873sup.zip › S0950268814001873sup004.doc]

*Epidemiology and Infection*

Effect of ethnicity on care pathway and outcomes in patients hospitalized with influenza A(H1N1)pdm09 in the UK

G. A. Nyland, B. C. McKenzie, P. R. Myles, M. G. Semple, W. Shen Lim, P. J. M. Openshaw, R. C. Read, B. L. Taylor, S. J. Brett, J. McMenamin, J. E. Enstone, B. Bannister, K. G. Nicholson, J. S. Nguyen-Van-Tam, on behalf of the Influenza Clinical Information Network (FLU-CIN)

Supplementary Material

## Supplementary Table S3 – Care pathway and clinical outcomes for patients admitted with influenza A(H1N1)pdm09 in the UK: major non-White ethnic sub-groups compared to White groups (n = 1140)

GP = general practitioner; OR = odds ratio (all comparisons with reference to White groups); CI = confidence interval; Adjusted OR (Model A) = adjusted for *a priori* confounders of age, sex, English Index of Multiple Deprivation (IMD 2007) score derived from postal code of residence and pandemic wave (* = also adjusted for admission delay ≥4 days and severity at presentation for admission); Adjusted OR (Model B) = adjusted for age, sex, IMD score, pandemic wave, recorded obesity, current smoking and chronic obstructive pulmonary disease (COPD); †One or more clinical indicators of severe disease at triage (see text); ‡Requiring high-dependency unit (HDU) or critical care unit (CCU); Percentages may not add to 100 due to rounding; Statistically significant results shown in bold (p<0.05)

| Variable | Crude OR (95% CI);  p value | Adjusted OR (95% CI);  p value (Model A) | Adjusted OR (95% CI);  p value (Model B) |
| --- | --- | --- | --- |
| Primary healthcare access indicators | | | |
| *Self-medication:* | | | |
| - White (n = 49; 7.8%) | 1.00 (reference) | 1.00 (reference) | 1.00 (reference) |
| - Asian/Asian British (n = 25; 10.0%) | 1.32 (0.80 – 2.19); 0.278 | **2.09 (1.09 – 4.00); 0.026** | **2.22 (1.14 – 4.30); 0.018** |
| - Black/Black British (n = 2; 1.6%) | **0.19 (0.04 – 0.78); 0.021** | 0.31 (0.07 – 1.39); 0.126 | 0.33 (0.07 – 1.50); 0.153 |
| - Chinese/mixed/others (n = 12; 9.1%) | 1.19 (0.61 – 2.30); 0.614 | 1.32 (0.61 – 2.85); 0.479 | 1.34 (0.62 – 2.93); 0.456 |

*Continues…*

| Variable | Crude OR (95% CI);  p value | Adjusted OR (95% CI);  p value (Model A) | Adjusted OR (95% CI);  p value (Model B) |
| --- | --- | --- | --- |
| Primary healthcare access indicators (continued) | | | |
| *GP consultation:* | | | |
| - White (n = 180; 28.6%) | 1.00 (reference) | 1.00 (reference) | 1.00 (reference) |
| - Asian/Asian British (n = 80; 32.1%) | 1.18 (0.86 – 1.63); 0.298 | 0.93 (0.62 – 1.38); 0.706 | 0.96 (0.64 – 1.45); 0.856 |
| - Black/Black British (n = 25; 19.4%) | **0.60 (0.38 – 0.96); 0.034** | **0.56 (0.32 – 0.96); 0.035** | **0.56 (0.32 – 0.98); 0.041** |
| - Chinese/mixed/others (n = 34; 25.8%) | 0.87 (0.57 – 1.33); 0.513 | 0.96 (0.58 – 1.57); 0.861 | 0.94 (0.57 – 1.54); 0.798 |
| *Pre-admission antibiotic:* | | | |
| - White (n = 142; 22.5%) | 1.00 (reference) | 1.00 (reference) | 1.00 (reference) |
| - Asian/Asian British (n = 30; 12.1%) | **0.47 (0.31 – 0.72); 0.001** | **0.49 (0.28 – 0.84); 0.010** | **0.51 (0.30 – 0.89); 0.017** |
| - Black/Black British (n = 9; 7.0%) | **0.26 (0.13 – 0.52); <0.001** | **0.40 (0.19 – 0.86); 0.019** | **0.42 (0.19 – 0.90); 0.025** |
| - Chinese/mixed/others (n = 15; 11.4%) | **0.44 (0.25 – 0.78); 0.005** | **0.37 (0.18 – 0.79); 0.009** | **0.40 (0.19 – 0.83); 0.015** |
| *Pre-admission antiviral:* | | | |
| - White (n = 68; 10.8%) | 1.00 (reference) | 1.00 (reference) | 1.00 (reference) |
| - Asian/Asian British (n = 31; 12.5%) | 1.18 (0.75 – 1.85); 0.484 | 1.01 ( 0.56 – 1.81); 0.978 | 0.96 (0.53 – 1.73); 0.890 |
| - Black/Black British (n = 7; 5.4%) | 0.47 (0.21 – 1.06); 0.068 | 0.41 (0.16 – 1.04); 0.062 | 0.39 (0.15 – 1.00); 0.050 |
| - Chinese/mixed/others (n = 13; 9.9%) | 0.90 (0.48 – 1.69); 0.749 | 1.05 (0.51 – 2.20); 0.887 | 1.02 (0.49 – 2.14); 0.952 |

*Continues…*

| Variable | Crude OR (95% CI);  p value | Adjusted OR (95% CI);  p value (Model A) | Adjusted OR (95% CI);  p value (Model B) |
| --- | --- | --- | --- |
| Secondary healthcare access indicators | | | |
| *Admission delay ≥4 days:* | | | |
| - White (n = 133; 21.1%) | 1.00 (reference) | 1.00 (reference) | 1.00 (reference) |
| - Asian/Asian British (n = 51; 20.5%) | 0.74 (0.51 – 1.09); 0.125 | 0.77 (0.48 – 1.26); 0.301 | 0.76 (0.47 – 1.25); 0.282 |
| - Black/Black British (n = 20; 15.5%) | **0.49 (0.29 – 0.83); 0.008** | 0.67 (0.36 – 1.25); 0.209 | 0.65 (0.35 – 1.21); 0.173 |
| - Chinese/mixed/others (n = 24; 18.2%) | 0.65 (0.40 – 1.07); 0.090 | 0.66 (0.36 – 1.22); 0.187 | 0.68 (0.36 – 1.26); 0.217 |
| *Severity at presentation:†* | | | |
| - White (n = 468; 74.3%) | 1.00 (reference) | 1.00 (reference) | 1.00 (reference) |
| - Asian/Asian British (n = 171; 68.7%) | 0.76 (0.55 – 1.05); 0.093 | 0.90 (0.60 – 1.33); 0.590 | 0.93 (0.62 – 1.39); 0.722 |
| - Black/Black British (n = 101; 78.3%) | 1.25 (0.79 – 1.97); 0.339 | 1.62 (0.95 – 2.75); 0.077 | 1.63 (0.96 – 2.78); 0.073 |
| - Chinese/mixed/others (n = 95; 72.0%) | 0.89 (0.58 – 1.35); 0.582 | 1.18 (0.71 – 1.96); 0.513 | 1.28 (0.77 – 2.12); 0.339 |

*Continues…*

| Variable | Crude OR (95% CI);  p value | Adjusted OR (95% CI);  p value (Model A) | Adjusted OR (95% CI);  p value (Model B) |
| --- | --- | --- | --- |
| In-patient care indicators | | | |
| *In-patient antibiotic:* | | | |
| - White (n = 513; 81.4%) | 1.00 (reference) | 1.00 (reference) | 1.00 (reference) |
| - Asian/Asian British (n = 213; 85.5%) | 1.35 (0.90 – 2.03); 0.148 | 1.66* (0.74 – 3.74); 0.220 | 1.89* (0.83 – 4.29); 0.129 |
| - Black/Black British (n = 113; 87.6%) | 1.61 (0.92 – 2.82); 0.096 | 1.85* (0.70 – 4.89); 0.214 | 2.07* (0.78 – 5.53); 0.146 |
| - Chinese/mixed/others (n = 114; 86.4%) | 1.44 (0.85 – 2.47); 0.179 | 1.56* (0.53 – 4.60); 0.418 | 1.72* (0.58 – 5.06); 0.327 |
| *In-patient antiviral:* | | | |
| - White (n = 452; 71.8%) | 1.00 (reference) | 1.00 (reference) | 1.00 (reference) |
| - Asian/Asian British (n = 187; 75.1%) | 1.19 (0.85 – 1.66); 0.315 | 1.41* (0.87 – 2.30); 0.167 | 1.50* (0.91 – 2.46); 0.112 |
| - Black/Black British (n = 103; 79.8%) | 1.56 (0.98 – 2.48); 0.060 | 1.81* (0.97 – 3.39); 0.064 | **2.03* (1.07– 3.85); 0.030** |
| - Chinese/mixed/others (n = 108; 81.8%) | **1.77 (1.10 – 2.85); 0.018** | **2.22* (1.16 – 4.26); 0.016** | **2.23* (1.16 – 4.31); 0.017** |

*Continues…*

| Variable | Crude OR (95% CI);  p value | Adjusted OR (95% CI);  p value (Model A) | Adjusted OR (95% CI);  p value (Model B) |
| --- | --- | --- | --- |
| Clinical outcomes | | | |
| *Length of stay ≥2 days:* | | | |
| - White (n = 427; 67.8%) | 1.00 (reference) | 1.00 (reference) | 1.00 (reference) |
| - Asian/Asian British (n = 163; 65.5%) | **0.68 (0.47 – 0.97); 0.034** | 0.95* (0.58 – 1.55); 0.824 | 1.04* (0.63 – 1.72); 0.866 |
| - Black/Black British (n = 96; 74.4%) | 0.95 (0.59 – 1.54); 0.839 | 1.11* (0.61 – 2.03); 0.730 | 1.22* (0.66 – 2.25); 0.524 |
| - Chinese/mixed/others (n = 98; 74.2%) | 1.05 (0.64 – 1.72); 0.841 | 1.60* (0.81 – 3.18); 0.176 | 1.75* (0.88 – 3.46); 0.110 |
| *Level 2 or 3 admission:‡* | | | |
| - White (n = 109; 17.3%) | 1.00 (reference) | 1.00 (reference) | 1.00 (reference) |
| - Asian/Asian British (n = 33; 13.3%) | 0.73 (0.48 – 1.11); 0.143 | 1.28 (0.75 – 2.21); 0.369 | 1.39 (0.80 – 2.42); 0.240 |
| - Black/Black British (n = 14; 10.9%) | 0.58 (0.32 – 1.05); 0.073 | 1.16 (0.57 – 2.34); 0.682 | 1.24 (0.61 – 2.54); 0.557 |
| - Chinese/mixed/others (n = 19; 14.4%) | 0.80 (0.47 – 1.36); 0.417 | 0.99 (0.48 – 2.02); 0.975 | 1.09 (0.53 – 2.25); 0.816 |
| *Death:* | | | |
| - White (n = 35; 5.6%) | 1.00 (reference) | 1.00 (reference) | 1.00 (reference) |
| - Asian/Asian British (n = 11; 4.4%) | 0.79 (0.39 – 1.57); 0.496 | 0.86 (0.33 – 2.20); 0.751 | 0.83 (0.32 – 2.15); 0.704 |
| - Black/Black British (n = 3; 2.3%) | 0.40 (0.12 – 1.34); 0.138 | 0.39 (0.08 – 1.84); 0.235 | 0.37 (0.08 – 1.75); 0.208 |
| - Chinese/mixed/others (n = 6; 4.6%) | 0.81 (0.33 – 1.97); 0.641 | 1.05 (0.32 – 3.38); 0.940 | 1.12 (0.34 – 3.70); 0.849 |

*End*
