# Supplementary material for: Effect of ethnicity on care pathway and outcomes in patients hospitalized with influenza A(H1N1)pdm09 in the UK
Source: Epidemiol Infect. 2014 Aug 1;143(6):1129–38. doi: 10.1017/S0950268814001873 (PMC4412072; doi:10.1017/S0950268814001873)
Supplement: Supplementary file 1 [file S0950268814001873sup.zip › S0950268814001873sup001.doc]

*Epidemiology and Infection*

Effect of ethnicity on care pathway and outcomes in patients hospitalized with influenza A(H1N1)pdm09 in the UK

G. A. Nyland, B. C. McKenzie, P. R. Myles, M. G. Semple, W. Shen Lim, P. J. M. Openshaw, R. C. Read, B. L. Taylor, S. J. Brett, J. McMenamin, J. E. Enstone, B. Bannister, K. G. Nicholson, J. S. Nguyen-Van-Tam, on behalf of the Influenza Clinical Information Network (FLU-CIN)

Supplementary Material

## Supplementary Figure S1 – FLU-CIN population mapped by postcode of residence showing (A) first wave cases ‘on top’ coloured yellow and (B) second wave cases ‘on top’ coloured red

(A)

*Continues…*

(B)

© 1988–2009 Microsoft Corporation and/or its suppliers. All rights reserved. http://www.microsoft.com/uk/mappoint/ © 1984-2009 Tele Atlas. All rights reserved. Data Source © 2009 Tele Atlas N.V. This product includes mapping data licenced from Ordnance Survey® with the permission of the Controller of Her Majesty’s Stationery Office. © Crown copyright and/or database right 2008. All rights reserved. Licence number 100025324. ©2009 NAVTEQ. All rights reserved. NAVTEQ ON BOARD is a registered trademark of NAVTEQ.

*End*
