# Supplementary material for: Effect of ethnicity on care pathway and outcomes in patients hospitalized with influenza A(H1N1)pdm09 in the UK
Source: Epidemiol Infect. 2014 Aug 1;143(6):1129–38. doi: 10.1017/S0950268814001873 (PMC4412072; doi:10.1017/S0950268814001873)
Supplement: Supplementary file 1 [file S0950268814001873sup.zip › S0950268814001873sup002.doc]

*Epidemiology and Infection*

Effect of ethnicity on care pathway and outcomes in patients hospitalized with influenza A(H1N1)pdm09 in the UK

G. A. Nyland, B. C. McKenzie, P. R. Myles, M. G. Semple, W. Shen Lim, P. J. M. Openshaw, R. C. Read, B. L. Taylor, S. J. Brett, J. McMenamin, J. E. Enstone, B. Bannister, K. G. Nicholson, J. S. Nguyen-Van-Tam, on behalf of the Influenza Clinical Information Network (FLU-CIN)

Supplementary Material

## Supplementary Table S1 – Community assessment tools (CATs) used for assessing illness severity at presentation for patients admitted with influenza A(H1N1)pdm09 in the UK by broad ethnic group (n = 1140)

OR = odds ratio; CI = confidence interval; Percentages may not add to 100 due to rounding; Statistically significant results shown in bold (p<0.05)

| CATs criterion | White  n = 630 (%) | Non-White  n = 510 (%) | Crude OR (95% CI);  p value |
| --- | --- | --- | --- |
| Criterion A (severe respiratory distress) | 292 (49.4) | 191 (37.5) | **0.69 (0.55–0.88); 0.003** |
| Criterion B (increased respiratory rate) | 177 (28.1) | 149 (29.2) | 1.06 (0.82–1.37); 0.677 |
| Criterion C (oxygen saturation ≤92% in air or on oxygen) | 233 (37.0) | 160 (31.4) | **0.78 (0.61–1.00); 0.048** |
| Criterion D (respiratory exhaustion or apnoeic episode) | 8 (1.3) | 7 (1.4) | 1.08 (0.39–3.00); 0.880 |
| Criterion E (severe clinical dehydration or clinical shock) | 81 (12.9) | 60 (11.8) | 0.90 (0.63–1.29); 0.578 |
| Criterion F (altered conscious level) | 38 (6.0) | 36 (7.1) | 1.18 (0.74–1.90); 0.484 |
| Criterion G (other clinical concern including rapidly progressive or unusually prolonged illness) | 80 (12.7) | 42 (8.2) | **0.62 (0.42–0.91); 0.016** |

*End*
